# On-chip, multisite extracellular and intracellular recordings from primary cultured skeletal myotubes

**DOI:** 10.1038/srep36498

**Published:** 2016-11-04

**Authors:** Noha Rabieh, Silviya M. Ojovan, Nava Shmoel, Hadas Erez, Eilon Maydan, Micha E. Spira

**Affiliations:** 1 Department of Neurobiology, the Alexander Silberman Institute of Life Science. The C. Smith Family and Prof. J. Elkes Laboratory for Collaborative Research in Psychobiology and the Harvey M. Kruger Family Center for Nanoscience. The Hebrew University of Jerusalem, Edmond J. Safra Campus, Jerusalem, 91904, Israel

## Abstract

In contrast to the extensive use of microelectrode array (MEA) technology in electrophysiological studies of cultured neurons and cardiac muscles, the vast field of skeletal muscle research has yet to adopt the technology. Here we demonstrate an empowering MEA technology for high quality, multisite, long-term electrophysiological recordings from cultured skeletal myotubes. Individual rat skeletal myotubes cultured on micrometer sized gold mushroom-shaped microelectrode (gMμE) based MEA tightly engulf the gMμEs, forming a high seal resistance between the myotubes and the gMμEs. As a consequence, spontaneous action potentials generated by the contracting myotubes are recorded as extracellular field potentials with amplitudes of up to 10 mV for over 14 days. Application of a 10 ms, 0.5–0.9 V voltage pulse through the gMμEs electroporated the myotube membrane, and transiently converted the extracellular to intracellular recording mode for 10–30 min. In a fraction of the cultures stable attenuated intracellular recordings were spontaneously produced. In these cases or after electroporation, subthreshold spontaneous potentials were also recorded. The introduction of the gMμE-MEA as a simple-to-use, high-quality electrophysiological tool together with the progress made in the use of cultured human myotubes opens up new venues for basic and clinical skeletal muscle research, preclinical drug screening, and personalized medicine.

Skeletal muscle research covers a wide range of basic and clinically oriented areas including muscle dystrophies and neuromuscular junction (NMJ) disorders such as myasthenia gravis, Lambert-Eaton myasthenic syndrome, amyotrophic lateral sclerosis, and spinal muscular atrophy SMA^1^,^2^,^3^,^4^,^5^,^6^. The passive and active membrane properties of skeletal muscles as well as the properties of the pre- and post-synaptic NMJ define many of the physiological properties of the skeletal muscle system. Both muscle membranes and the NMJs are the targets of a wide range of genetic pathologies. Thus, electrophysiological characterization of skeletal muscles and NMJs during development in health and disease is essential.

In contrast to the extensive use of substrate integrated planar multielectrode arrays (MEA) to characterize the electrophysiological properties of cultured neuron and cardiac muscles networks^7^,^8^,^9^,^10^,^11^,^12^,^13^,^14^ the vast community of skeletal muscle researchers do not make use of MEA. One plausible reason why MEA technology has not been adopted for skeletal muscle research and diagnostics is because progress in the culturing technologies of skeletal myotubes and NMJ from rodents and human is relatively recent^15^,^16^,^17^,^18^,^19^,^20^. To date, only two original studies have been published on the use of substrate integrated planar MEA to electrophysiologically record from cultured skeletal myotubes^21^,^22^.

It is important to note that currently *in vitro* preclinical drug screening as well as characterizations of skeletal muscle development and pathophysiology rely mainly on imaging of muscle contraction dynamics and associated alterations in their free intracellular calcium concentrations ([Ca^2+^]_i_)^15^,^18^,^20^,^23^,^24^,^25^,^26^. The monitoring of muscle contraction dynamics or [Ca^2+^]_i_ imaging cannot provide the information obtained by electrophysiological recordings since [Ca^2+^]_i_ imaging reflects the integral of complex processes of Ca^2+^ influx through voltage gated calcium channels, voltage dependent or independent release of Ca^2+^ from intracellular stores, and the removal dynamics of Ca^2+^ by a very large number of mechanisms. Alterations in muscle contraction dynamics during development or by pathological conditions also provide an integrated output of a large number of complex cellular mechanisms; thus monitoring contraction dynamics concomitantly with [Ca^2+^]_i_ is still insufficient and could greatly benefit from complementary electrophysiological readouts^27^.

In recent years, our laboratory has developed a novel approach to multisite, non-invasive recordings electroactive cells dubbed “IN-CELL” recording. In this method micrometer-sized, extracellular gold mushroom-shaped microelectrodes (gMμEs) record attenuated synaptic and action potentials (APs) with characteristic features of intracellular recordings while the electrode maintains an extracellular position^28^,^29^,^30^,^31^,^32^,^33^,^34^,^35^. These studies show that a range of cell types tightly engulf gMμEs to form high seal resistance (*R*_*s*_)^29^,^30^,^36^,^37^,^38^. This, together with the increased conductance of the neuronal membrane that faces the electrode (the junctional membrane – *jm*), makes it possible to record from cultured *Aplysia* neurons APs and subthreshold synaptic potentials with qualities and biophysics similar to perforated patch recordings^39^,^40^. Analyses of the neuron-gMμE junction have indicated that in fact the physical principles that enable the “in-cell recording” configuration are identical to those used in the perforated patch electrode configuration^39^,^40^.

In the present article we present a gMμEs based multielectrode array (gMμE-MEA) to monitor the electrophysiological properties of cultured skeletal myotubes. The introduction of the gMμE-MEA as an simple-to-use, electrophysiological tool to record subthreshold- and action-potentials from post synaptic skeletal myotubes complemented by the progress made in the co-culture of motoneurons and myotubes to form functional NMJ and the possible use of cultured embryonic and human induced pluripotent stem cells is expected to open up new venues for basic and clinical skeletal muscle research, preclinical drug screening, and personalized medicine. In addition, gMμE-MEA platforms can be used to improve recordings from skeletal muscles that have been re-innervated by nerves that lost their targets after limb amputation. The *in vivo* re-innervated surgically reconstructed muscles can then serve as biological amplifiers that relay electrical signaling via a MEA platforms to robotic arms^41^.

## Results

### Structural relationships between cultured skeletal myotubes and gold mushroom-shaped micro-protrusions

**Figure 1:**
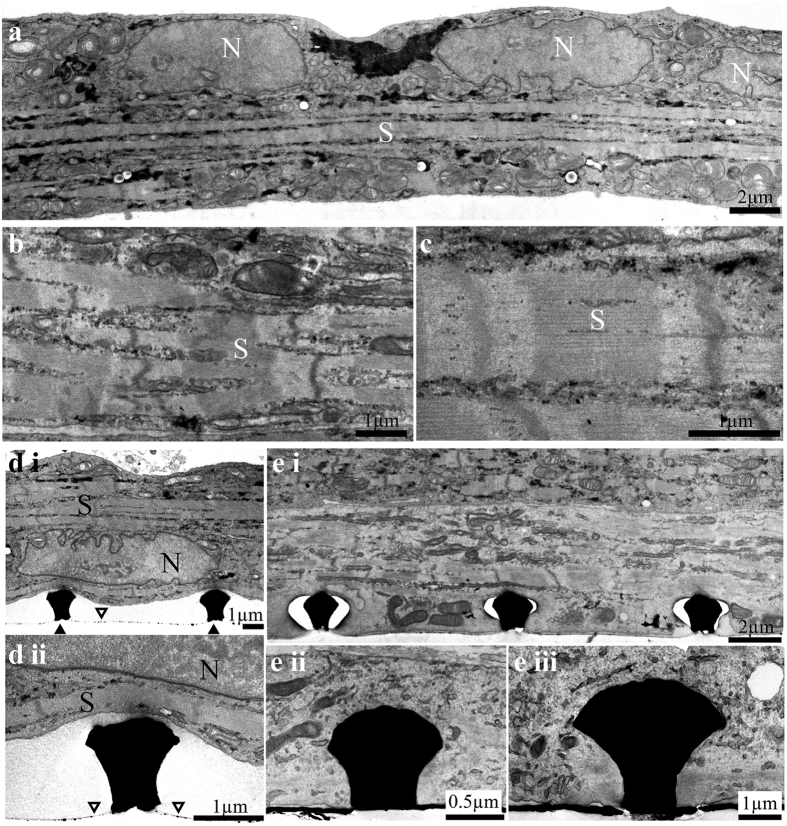
Transmission electron micrographs of 10 day old cultured myotubes. (**a**) Low magnification of a long multinucleated myotube (3 nuclei –N) with a distinct region showing sarcomere structures (S). The sarcomeres are enlarged in (**b**,**c**). (di and dii) a cultured myotube “resting” on top of gMμPs (black arrowheads) does not adhere to the culture substrate that is spattered with residual small gold particles (empty arrow heads). (**e**) Example of the engulfment of gMμPs by cultured myotubes. (ei) low magnification of a long myotube engulfing three gMμPs. The translucent areas around the gMμPs were formed by the electron beam of the microscope during the observation. (eii and eiii), examples of tight engulfment of gMμPs by myotubes which also adhere to the gold substrate (black).

To examine whether cultured skeletal myotubes can engulf gold mushroom-shaped protrusions (gMμP) and thereby generate the structural substrate for high seal resistance formation we plated myoblasts on high density gMμP matrices with geometric features identical to the gMμE that were fabricated for electrical recordings. The high density gMμP matrices with 8 μm inter gMμP interval were used to increase the probability of collecting thin sections that run through myotubes and gMμP for transmission electron microscope (TEM) imaging. TEM imaging of 6–10 day old cultured myotubes revealed multinuclear myotubes characterized by typical acto-myosin striations (Fig. 1) and fibroblasts^42^ (here and in other parts of the manuscript we refer to the days in culture by counting the number of days since the last replating cycle of the myoblast on gMμP matrices or gMμE-MEA). Whereas a fraction of the myotubes and fibroblasts resided on top of the gMμPs and did not adhere to the flat substrate in between the gMμP (Fig. 1di,dii), other fractions of the myotubes engulfed the gMμPs and adhered to the flat substrate around them (Fig. 1ei–eiii).

In the first case the cleft formed between the myotubes’ plasma membrane and the gMμP surface was in the range of 200–500 nm. In the second case, the plasma membrane tightly adhered to the entire surface of the gMμPs and to the flat substrate around it (Fig. 1e). The well preserved appearance of the subcellular organelles in the TEM images suggested that the fixation, dehydration and embedding procedures did not produce significant ultrastructural artifacts. Nevertheless, it is important to recall that the extracellular cleft formed between the plasma membrane of living cells and artificial substrates such as the gMμPs may reflect varying degrees of artifacts induced by the processing of the samples for TEM analysis^35^,^43^. A number of studies have estimated that the processing of tissues for TEM imaging induces “shrinking artifacts” in the range of 5–17%^44^,^45^. Thus, it is possible that the direct contact imaged between the myotube and the gMμP (Fig. 1e) is due to shrinkage.

In summary, the structural observations (Fig. 1) revealed that under the culturing protocols used in the present study, the gMμP matrix and gMμE-MEA provided a bio-compatible substrate for the unstructured development of myotubes and that the interfaces formed by the developing myotubes and gMμP matrix ranged from loose to tight adhesion.

### Electrical recordings from cultured myotubes by gold mushroom based MEA

**Figure 2:**
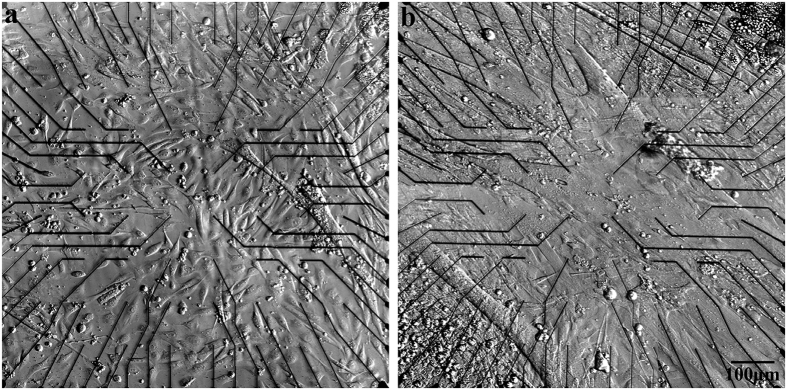
Skeletal myotubes cultured on polydopamine and matrigel functionalized gMμE-MEA. (**a**) 1DIV and (**b**) 4 DIV after the final plating cycle.

**Figure 3:**
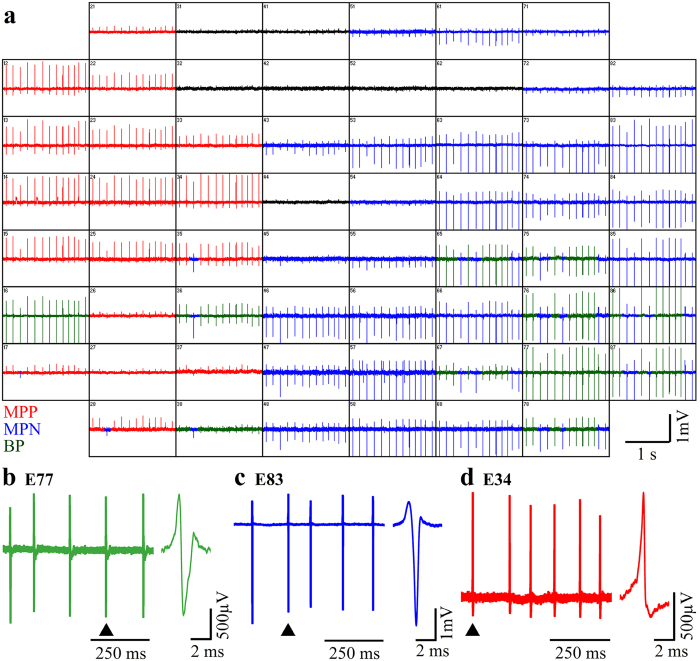
Raw recordings of spontaneous field potentials by gMμE-MEA from cultured myotube 4 DIV. Each box show FPs recordings by a single gMμE. Biphasic field potentials are depicted in green (**a**) and enlarged in (**b**, electrode 77). FPs dominated by a negative component are labeled in blue, and enlarged in (**c**, electrode 83) and FPs dominated by a positive component are labeled red, and enlarged in (**d**, electrode 34). Electrodes that peaked up FPs <0.1 mV are marked in black. Note that a fraction of the gMμE record both negative and biphasic FPs and others both negative and positive FPs. Whereas the FPs waveform shape (**c**) suggests that the majority of the individual gMμE pick up the activity from a single myotube, it is interesting to note that the FP amplitudes recorded by individual gMμE were not constant.

To characterize the electrophysiological signaling repertoire generated by cultured myotubes, 3–14 DIV replated myoblasts on functionalized gMμE-MEA were used. At this stage the myoblasts fused to form elongated and sometimes bifurcating myotubes^46^ (Fig. 2) that began to spontaneously contract. Contractions were associated with recordings of FPs with peak to peak amplitudes of 0.1 mV up to 10 mV (Fig. 3). The FPs could be classified as biphasic, negative, or positive dominated potentials in a similar manner to those recorded by extracellular electrodes from non isopotential neurons or cardiomyocytes^34^,^47^,^48^. Based on the “simple” shape of the FPs, their amplitude, and the fact that the dimensions and surface area of the gMμE are very small (~10 μm^2^) with respect to the dimensions of the myotubes, it is reasonable to assume that most individual gMμE recorded FPs from a single myotube (Fig. 3b–d). A small fraction of recorded individual gMμEs comprised FPs that were most likely generated by a number of adjacent myotubes. It should be noted that the average amplitudes of the FPs recorded by the gMμE-MEAs were significantly larger than those reported in two earlier publications by Langhammer *et al*.^21^,^22^, using substrate integrated planar electrode based MEA with a surface area of **314 **μm^2^ 21,22. Whereas the average FP amplitude recorded by Langhammer *et al*. was in the range of 100 μV, in the present study the average amplitudes for positive monophasic FPs were 1279 ± 146 μV (n = 148); for negative monophasic FPs 711 ± 66 μV (n = 45) and for the biphasic FPs 1221 ± 1045 μV (n = 557). The improved recording quality of the gMμE-MEA over substrate integrated planar MEA can be attributed to the increased seal resistance formed between the myotubes’ plasma membrane and the gMμE by its engulfment^29^,^30^,^31^,^32^,^43^,^48^. Consistent with this hypothesis, the variability in the FP amplitudes is related to the level of gMμE engulfment by the cells^43^.

**Figure 4:**
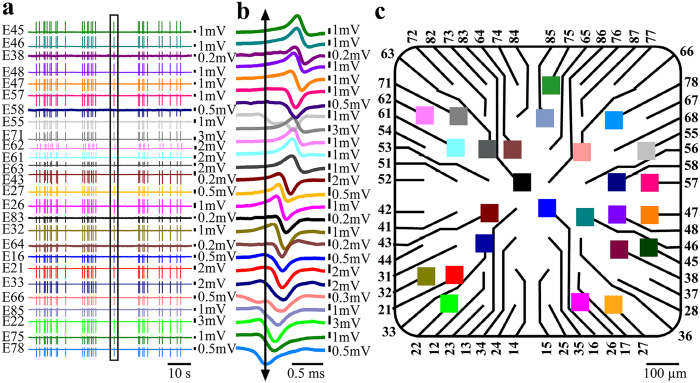
Concomitant bursts of field potentials recorded from a population of myotube 7 DIV after the final replating. (**a**) Raw FP recordings from 26 gMμE reveal that gMμE spaced over the entire recording surface area (total recording surface of 0.8 × 0.8 mm) fire in apparent synchrony. Enlargement of the FPs marked by a box in (**a**) shows that the FPs recorded by the different gMμE have different waveform shapes (**b,c**) and are not generated at the same time (**c**). The FPs propagates at an estimated rate of 150–200 cm/s. This may represent the conduction velocity of action potentials along large myotubes or that a fraction of the 7 DIV myotubes were electrically coupled.

In contrast to the expected electrical independence of mature myotubes^21^ in most cultures, concomitant spontaneous bursts of FPs were recorded from day 3–4 and onward after the last replating cycle by the gMμE (Fig. 4a). Examination of the temporal relationships between the FPs recorded by different electrodes on a fast time scale (Fig. 4b) revealed that some FPs within a burst were almost perfectly synchronized (Fig. 4b electrodes: 45, 46, 38 and electrodes 64, 16 and 21) others revealed that the FPs propagated at rates of 150–200 cm/s along the culture (Fig. 4c for electrode distribution map). Whereas the nearly synchronized FPs (with a jitter of ~100 μs) was probably recorded by gMμEs that contacted the same myotube, other FP may have propagated across myotubes in the culture at slower rates. This observation implies that at this stage the myotubes in our *in vitro* cultures are electrically interconnected, possibly by unfinished myotube fusion processes. Interestingly, the propagation rates of the action potentials along electrically coupled cardiomyocytes in culture was reported to be slower, in the range of 40 cm/sec^49^.

In spite of the continuous contractions of the myotubes large FP were continuously recorded over a period of 10–14 days. This implies that the spatial relationships between the contracting myotubes and individual gMμE were stable for this period. Thereafter the number and amplitude of the recorded potentials diminished in association with myotubes detachment from the substrate and rounding up.

Furthermore, whereas the shape of the FPs recorded by individual gMμE remained constant during a recording sessions which lasted for ~30 minutes, the amplitudes of the FPs varied intermittently by 10–20% (Fig. 3b–d). These relatively large fluctuations in the FP amplitudes cannot be attributed to the noise level of the system since it was significantly smaller. The possible source of this variability is discussed below.

### Intracellular recordings of action potentials by gMμE-MEA

The ultrastructural observations described above revealed that a fraction of the gMμE-MEA were tightly engulfed by cultured myotubes in a similar manner to that described by our laboratory for cultured *Aplysia* neurons, cardiomyocytes and rat hippocampal neurons^28^,^29^,^30^,^31^,^32^,^33^,^34^,^43^. The tight engulfment of the gMμEs by myotubes provides the necessary structural basis to obtain Ohmic access to the myotube sarcoplasm and IN-CELL recording configurations.

**Figure 5:**
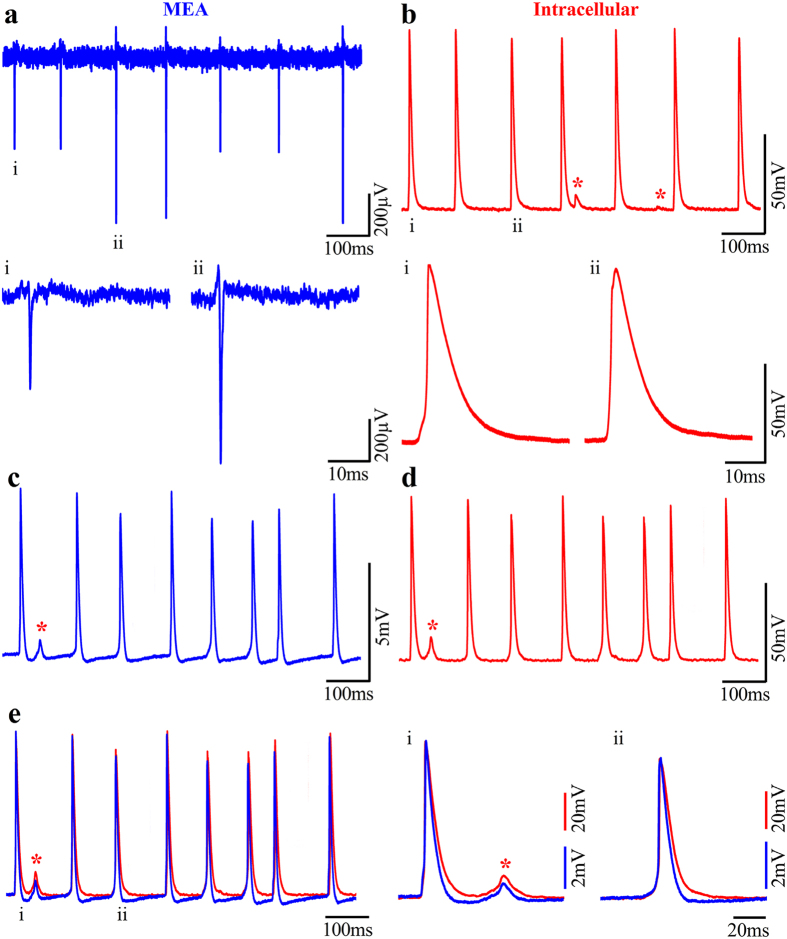
Comparison of intracellular recorded potentials to IN-CELL recordings from cultured myotubes. Concomitant spontaneous extracellular FPs recordings by a gMμE (**a**) and intracellular recordings by sharp electrodes (**b**) from 3 DIV myotubes after the final replating. The recordings in (**a**,**b**) revealed identical firing patterns and similar qualitative alterations in the amplitudes of the recorded action potentials. The field potentials labeled i, and ii, and action potentials i and ii are shown in the lower traces with an expanded time scale. (**c**) Electroporation of the myotube changed the gMμE mode of recording from extracellular to the IN-CELL mode. Note that although the recorded amplitude of the spikes is about an order of magnitude lower than that of the intracellular electrode, the shape of the recorded potentials are identical. Also, note that in (**c,d**) both electrodes recorded subthreshold potentials (red asterisk) in between the spikes. (**e**) Merge traces of c (blue) and d (red), i and ii are traces with an expanded time scale.

**Figure 6:**
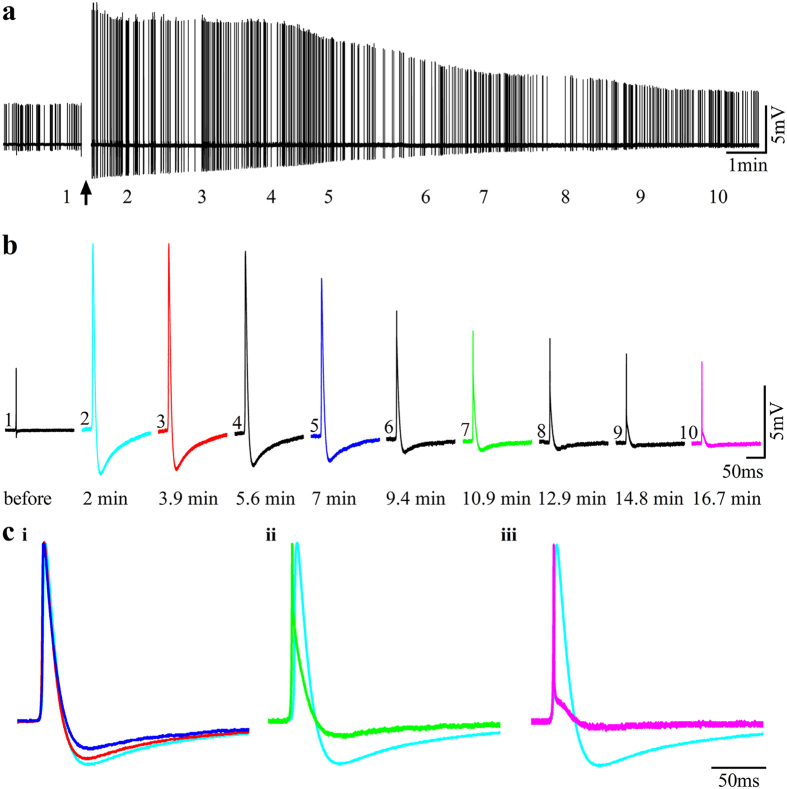
Accessing IN-CELL recording by membrane electroporation and its reversal by membrane repair (3DIV after the final replating). (a1) Before electroporation, a gMμE recorded an extracellular positive FP (a1 and b1) characterized by 3.6 mV and a short duration of 0.25 ms (50% height). After the delivery of an electroporating pulse, the extracellular FP transformed into a 12 mV, 4.9 ms. IN-CELL recorded potential. The amplitude of the action potential gradually diminished over a period of approximately 30 minutes (**a,b**), resuming the shape of an extracellular field potential (ci–iii). Super-positioning of the first IN-CELL recorded potential (light blue) on normalized potentials recorded at different points in time after the electroporation (color coded as in **b**) revealed that aside from a gradual reduction of the spike amplitude shown in (**b**) the duration of the potential was gradually and significantly reduced as the recording configuration changed from IN-CELL to extracellular (**c**).

Ohmic access to the sarcoplasm by gMμEs can be generated either by electroporation or spontaneously. Application of 10 ms long, 500–900 mV positive square pulses through gMμEs led in over 50% of the experiments (19/34 MEA) to membrane electroporation and the transition from an extracellular FPs recording mode to an intracellular recording of attenuated APs with amplitudes ranging from 2 to 10 mV (Figs 5 and 6). Intracellular recording of action potentials following electroporation lasted up to ~30 minutes (Fig. 6). Thereafter, cell biological repair processes sealed off the electroporated nanopores^50^ and the intracellularly recorded action potential gradually reverted to the typical shaped extracellular FP (Fig. 6)^34^,^51^.

Examples of the switch from extracellular to attenuated intracellular recordings by electroporation and the recovery to the extracellular recording mode are illustrated in Figs 5 and 6. When the gMμE recorded negative FPs (Fig. 5) the transition to intracellular recording was associated with the reversal of FPs polarity to monophasic positive AP, as well as an increase in the AP amplitude and duration (Fig. 5). The increase in the AP amplitude and duration reflects the reduction in the junctional membrane resistance^35^. The recovery process progressed by reduction in amplitude and by a gradual narrowing of the waveform, as illustrated by the super-positioning of normalized spike amplitudes at different points in time during the recovery period (Fig. 6c, light blue).

To compare the quality of the attenuated intracellular recordings obtained by the gMμE-MEA to those obtained by intracellular recordings with sharp intracellular glass microelectrodes we inserted a glass microelectrode into a myocyte while recording its spontaneous activity by a gMμE (Fig. 5a,b). Then we applied an electroporating pulse through the gMμE and recorded the spontaneous electrical activity by both the gMμE and the glass microelectrode (Fig. 5c,d). Notably, whereas the amplitudes of the APs recorded by the gMμE were about a tenth of that recorded by the intracellular glass electrode, the shapes of the recorded potentials were similar (Fig. 5e). The small differences in the shapes of the APs can be ascribed to the filtering effects of the gMμE and the AC MEA amplifiers and the DC amplifier used for recording the potentials by the sharp glass electrodes^33^,^35^,^43^,^52^.

Aside from the spontaneous APs, both the intracellular sharp electrodes and the gMμE recorded small subthreshold potentials which at first glance resembled excitatory postsynaptic potentials (Fig. 5 red asterisk). Because the cultures from which these types of subthreshold potentials were recorded are devoid of neurons, these subthreshold potentials could not represent chemical synapses. We suggest that these subthreshold potentials represent the electrotonic spread of AP among partially fused neighboring electrically coupled myotubes or the electrotonic spread of action potentials at points of cell bifurcations from a narrow myotube compartment to a large compartment as discussed below.

### The source of the observed subthreshold potentials and alterations in spike amplitudes

Both intracellular recordings by sharp glass electrodes and the gMμE-MEA revealed two unusual phenomena: (a) the recordings of spontaneous subthreshold potentials in the absence of neuronal elements in the culture and (b), 10–20% alterations in the amplitudes of the spontaneously recorded intracellular AP and FP (Fig. 5). Both phenomena are consistent with the hypothesis that the fusion of myoblasts to form myotubes and the secondary fusion of myotubes in 3–14 day old unstructured cell cultures is incomplete.

**Figure 7:**
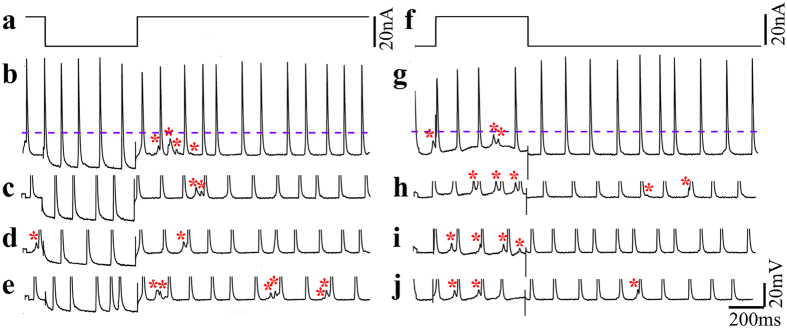
Spontaneous subthreshold potentials are generated by the spread of current generated among electrically coupled myotubes. Intracellular recordings from spontaneously firing myotubes (3 DIV after the final plating cycle) cultured in the absence of neurons were made by a sharp intracellular microelectrode. The electrode was used for both current injection and voltage recordings. (**a**) hyperpolarizing square current pulse injunction. Traces of spontaneous firing (**b–e**) during hyperpolarization of the myotube from which the recordings were made reduced the frequency of the subthreshold potentials. In (**b**) spontaneous spikes and subthreshold potentials (red asterisks) are shown whereas in (**c–e**) the recordings were trimmed along the dashed line shown in (**b**). On the other hand depolarization (**f**) increased the frequency of the subthreshold potentials (**g–j** red asterisk). In (**g**) spontaneous spikes and subthreshold potentials (red asterisks) are shown whereas in (**h–j**) the recordings were trimmed along the dashed line as shown in (**g**).

Accordingly it is assumed that the current generated by the firing of APs by a single myotube passively spreads to neighboring electrically coupled myotubes but is insufficient to reach the firing threshold of the neighboring myotubes. The above hypothesis is supported by the observation that the subthreshold potentials behave as electrical postsynaptic potentials. Thus, hyperpolarization of the myotube (from which both action potentials and subthreshold synaptic potentials were recorded) by intracellular current injection (through the glass microelectrode) did not lead to an increase in the amplitude of the subthreshold potentials (Fig. 7a–e, red asterisk). Similarly, depolarization of the myotube from which both APs and the subthreshold potentials were recorded did not lead to a decrease in its amplitude (Fig. 7f–j). In addition, hyperpolarization of the myotube by current injections through the glass electrode led to a reduction in the frequency of the recorded subthreshold potentials and depolarization increased their frequency (Fig. 7a–e).

An alternative explanation to the observation could be related to the complex bifurcating geometry of myotubes and heterogeneous diameters of a single myotubes grown on an un-patterned substrate^46^. It is well established that action potentials fail to propagate across points of impedance mismatching along different compartments of the same neurons. For example, antidromically propagating APs fail to invade the cell body as an outcome of the sudden increase in the surface area of the cell body in relationship to the axons or along axons with a heterogeneous diameters. Failure of AP propagation was also found at points of axonal bifurcations. In these cases the AP voltage spread from the point of failure as a subthreshold potential^53^,^54^,^55^. Although the structural basis underlying the failure of AP to propagate in the case of electrically coupled myotubes and along point of myotube bifurcations is different, the underlying biophysical mechanisms are very similar.

**Figure 8:**
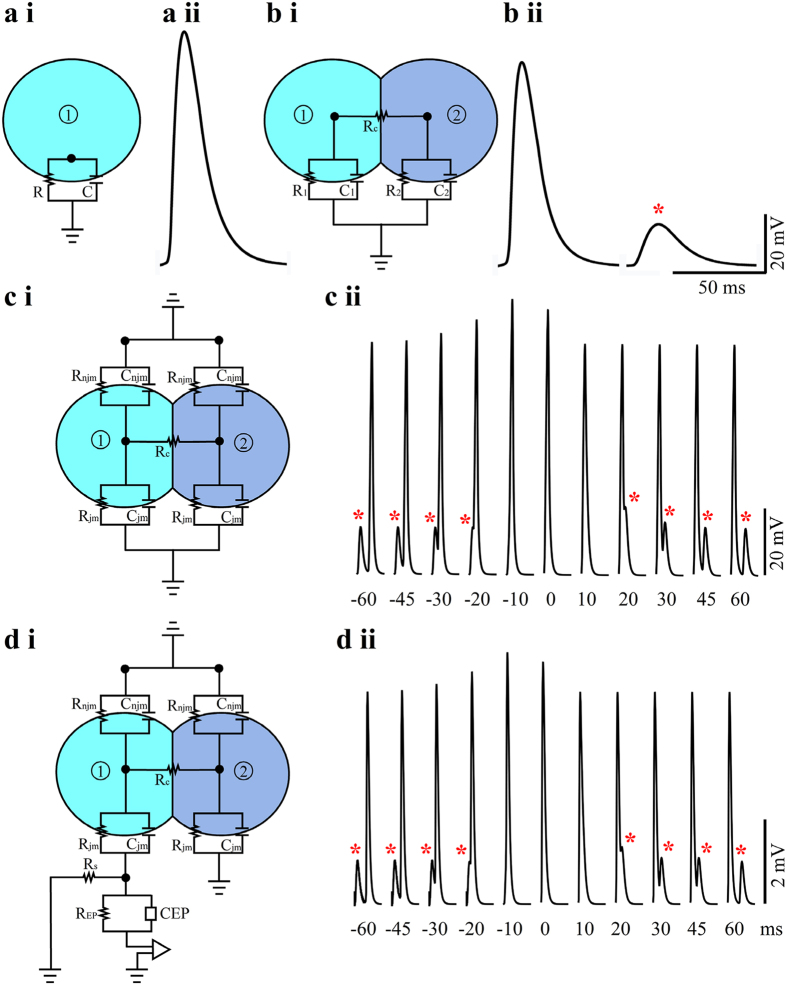
Simulation of action potential amplitudes and their electrotonic spread among coupled myotubes. (aii) An 80 mV action potential generated by myotube spike current pulse injection (2.22 nA) into an isolated model myotube (as shown in ai). (bii) When the same current pulse (2.22 nA) is injected into the same myotube but after its coupling to a second myotube (as shown in bi) the voltage amplitude is reduced to 70 mV. Concomitantly (cii), an attenuated potential-an electrotonic excitatory potential is recorded in the second coupled myotube (red asterisk). Action potential and voltage spread recorded “intracellularly” from myotube 1 when an action potential current is injected first into myotube 2 and then into myotube 1 (ci). The time interval between the two current injections is reduced from −60 to 0 ms (in 10 ms steps) and then increased to + 60 ms. As the decremental potential (red asterisk) and the action potential summate, the peak amplitude of the action potential is increased and reaches a maximum at an interval of −10. (dii) The attenuated potentials as “recorded” by a gMuE (di). Note that although the shapes of the simulated potentials are similar, the interface with a gMμE simulation circuit leads to attenuation of the potentials’ amplitudes.

Electrical coupling among myotubes and the bifurcating morphology of the myotubes could also account for the rather unusual observations of alterations of the intracellularly recorded APs amplitudes (Fig. 5). This rather unusual behavior was further illustrated and examined by the use of the SPICE simulation system (Tanner EDA v.15) of a simplified case involving two passive analog electrical circuits depicting two coupled isopotential cells (Fig. 8) or two compartments of the same myotube.

For the simulation we fed a current pulse with a shape of a recorded action potential into an “isolated myotube” (Fig. 8a). The current amplitude was adjusted to generate an 80 mV peak voltage across a single isolated “myotube membrane” with an input resistance of 100 MΩ (R1 = 100 MΩ, C1 = 100pF, Fig. 8a). When the very same current pulse was injected into myotube 1, after it was electrically coupled to “myotube 2” (R2 = 100 MΩ. C2 = 100pF) by a coupling resistor Rc of 250 MΩ (Fig. 8b) the AP amplitude recorded in cell 1 diminished by 10 mV from 80 to 70 mV (Fig. 8b). Concomitantly, the current injected into myotube 1 spread into myotube 2 to form an electrical post synaptic -like potential with an amplitude of 10 mV (Fig. 8c). To illustrate the contribution of the current spread from one myotube to the other on the recorded APs amplitudes we next simulated the voltages in cell one, when cells 1 and 2 fired at different time intervals. Fig. 8c illustrates the expected “recordings” from myotube 1 when myotube 2 fired before, together and after myotube 1. It can be seen that as a result of voltage summation between the action potential and the electrotonic spread, the amplitude of the APs amplitude varied by approximately 10 mV.

Because the gMμE recording system attenuates and filters the recorded potentials we next repeated the simulation of the recording shown in Fig. 8d with a myotube-gMμE circuit^43^. The parameters used were those applied in the simulation of Fig. 8d with the addition of a circuit depicting the engulfment of a gMμE by myotube 1. As shown in Fig. 8d, in terms of the shape and relative amplitudes, the distortions introduced by the gMμE-MEA “recordings” were negligible.

It is noted that the 50% variability in the amplitudes of the extracellular FPs within a given train of APs (Fig. 5a) is much larger than the 10–20% differences observed for the intracellularly recorded APs (by a sharp glass microelectrode) or the amplitudes of the IN CELL recorded APs (Figs 5b–d and 8). Recall that the extracellular FPs represent the time derivative of the intracellular recorded potentials. Therefore, the amplitude of the extracellular FP is very sensitive to the rise- and decay times of the intracellular AP (Supplementary Fig. S1).

## Discussion

The main finding of this study is that gMμE-MEA platforms can be effectively implemented in basic and applied skeletal muscle research and thereby contribute an important electrophysiological technique to this field. This, together with the recent progress in the use of cultured human myotubes and the formation of NMJ *in vitro* is expected to open up new venues for basic and clinical research, preclinical drug screening, and personalized medicine. In contrast to the extensive use of substrate integrated MEA devices in neuroscience and cardiology research only two published papers have examined the use of MEA for *in vitro* skeletal myotube research. In these studies Langhammer *et al*.^21^,^22^ demonstrated that substrate integrated planar MEA, with a surface area of 314 μm^2^, record multiphasic spontaneous FPs from rat cultured myotubes with an average amplitude of 100 μV. Analysis of the recorded FPs suggested that even when grown on unstructured substrate myotubes undergo the essential developmental cascades to become electrophysiologically independent of each other. The electrical independence of myotubes is a critical feature that enables skeletal muscles to generate graded and controlable generation and contraction dynamics^21^,^22^.

In comparison to the substrate integrated planar MEA studies by Langhammere *et al*.^21^,^22^, the gMμE-MEA provides significantly improved source resolution of individual myotubes and larger FPs (Figs 3 and 5). Furthermore, in response to electroporating pulses delivered through gMμEs, the myotube-gMμE recording configuration can be switched from an extracellular to attenuated intracellular recording mode (Fig. 5). Under these conditions the shape and duration of the recorded action potentials by the gMμE (but not the amplitude) are similar to those recorded by sharp intracellular microelectrodes for minutes (Fig. 6.). As in other cell types, after electroporation, cell biological processes repair the electroporated membrane and revert the recording mode back to extracellular (Fig. 6)^34^,^51^,^52^,^56^,^57^. It is conceivable that functionalization of the gMμEs with alkanethiols might facilitate the formation of a stable high seal resistance junction between the electrode and the cell’s plasma membrane^58^,^59^,^60^,^61^. Under such conditions the gMμE may be in direct contact with the neuron’s cytosol for long periods of time^52^,^58^,^62^. In a small fraction of the experiments (5%) spontaneous IN-CELL recordings were observed to form between the myotubes and the gMμEs. In these cases the IN-CELL recording configuration was stable for at least the duration of the recording session which lasted 30–45 min. As previously shown and discussed, IN-CELL recordings are the outcome of a combination of factors including the seal resistance formed by the engulfment of a gMμE by a cell, the junctional membrane conductance (the patch of membrane that faces the gMμE) of the cell, and the electrode impedance^14^,^31^,^32^,^33^,^35^,^63^. Although spontaneous IN-CELL recordings of myotubes are rare it is conceivable that methods to facilitate their spontaneous formation can be developed. For example, this can be achieved by the use of chemical functionalization of the gMμE that locally increases the adhesion between the plasma membrane by chemical functionalization of the gold mushrooms^64^ and increases the junctional membrane conductance^31^,^32^.

It is of interest to note that intracellular (Fig. 7) or IN-CELL (Fig. 5) recordings from myoblasts revealed the emergence of spontaneous subthreshold potentials in between the full blown spikes. As the culture does not contain any neurons it is possible to unequivocally define these potentials as decremented action potentials that passively spread along or among myotubes. The shape and properties of these subthreshold potentials resemble those of electrical synaptic potentials, as documented in electrically coupled neurons and cardiomyocytes^65^, or the electrotonic spread of action potentials at points of cell bifurcation^53^,^54^,^55^. Thus, these subthreshold potentials could be generated by current spread between partially fused myotubes or at points of bifurcations of a single myotube^46^. The electrical behavior in both cases is expected to be similar and follows the principles depicted in the analog electrical circuit in Fig. 8. Recall however, that the simplified passive analog electrical circuit used here illustrates only two electrically coupled myotubes or two connected cellular compartments (branches) of the same cell, whereas in culture a number of myotubes may be electrically coupled to each other or one myotube can extend a number of branches^46^. As revealed by the simulation of Fig. 8 the passive load of electrically coupled myotubes, or the myotube branches that do not fire synchronously may be sufficient to reduce the AP amplitude measured intra- or extra-cellularly (Fig. 5). Synchronized or nearly synchronized firing of coupled myotubes or cell compartments (branches) reduces the electrical load and makes it possible to record full blown APs. Here we did not discriminate between the two possible mechanisms. In either case the observation suggests that under the culture conditions used by us the myotubes are either not isolated electrically from each other or the branching of individual myotubes grown on unstructured substrate leads to pathological electrophysiological behavior that is expected to effect the dynamic behavior of single myotube contractions.

Based on the present study it is apparent that IN-CELL recording from myotubes could be used to record excitatory postsynaptic potentials from NMJ formed by co-culturing motor neurons and skeletal myotubes. This, together with the recent development of biotechnologies to use induced human pluripotent stem cells from healthy human subjects and patients, will enable the study of NMJ disease mechanisms, *in vitro* drug screening on human cells and the development of personalized medical procedures^15^,^16^,^17^,^18^,^19^,^20^,^66^,^67^.

Another potential application of the gMμE-MEA is for recordings from skeletal muscles that have been re-innervated by nerves that lost their targets after limb amputation. Recordings from surgically-reconstructed muscles can serve as biological amplifiers that relay electrical signaling via the gMμE-MEA platforms to prosthetic arms^41^,^68^,^69^.

In conclusion we demonstrated that the gold mushroom-shaped microelectrode based MEA can serve as a convenient tool for simultaneous, extracellular or intracellular recordings from cultured myotubes. Using standard fabrication procedures, the gMμE-MEA can be integrated with poly(dimethylsiloxane) (PDMS) structures to form multiple compartment MEA-devices. This can be used for co-culturing motor neurons and myotubes to form a convenient setting to study the development and pharmacology of NMJ, drug screening, the study of different muscular dystrophies, muscle injury and the development of personalized medicine.

## Methods

### Fabrication of gold mushroom-shaped micro protrusions matrices for electron microscopy and gold mushroom-shaped microelectrode array

Gold mushroom microprotrusions (gMμP) matrices and gMμE-MEA were manufactured as described earlier by our laboratory^33^.

Chips of dense gMμP were prepared on 200 μm glass wafers (AF45 Schott Glass) by means of photolithography and electroplating techniques. Briefly, the wafers were coated with a 60 nm Au layer by thermal evaporation on top of a 10 nm Ti layer (e-gun evaporation), spin-coated with Shipley photoresist S1813 and 120 °C hard bake for 10 min. The photoresist layer was then exposed to UV using a photomask with 1 μm holes with a pitch of 8 μm (Karl Suss MicroTec MA6 mask aligner, UV 365 nm W = 26 mW/cm^2^, exposure time: 2.7 s). AZ726 for 35 s was used for development. gMμPs were then electroplated using Neutronex gold plating solution. The wafer was diced, cleaned and The gMμP matrices chips were glued to the bottom of conventional plastic culture dishes.

gMμE-MEA were prepared on 300 μm thick glass wafers (AF45 Schott Glass) by means of photolithography and electroplating techniques. Briefly, the wafers were coated with a Ti (10 nm)/Au layer (100 nm) by way of thermal evaporation, spin-coated with photoresist AZ-1505 (4,000 RPM) and hard baked for 2 min (120 °C). Thereafter a photolithographic process to define the conducting lines was performed by wet etching of the Ti/Au in between the conducting lines. Next, a lithographic step using Shipley S-1813G2 photoresist (4,000 RPM) hard baked was performed to open up 1 μm holes for the electro deposition of the gMμE-stalks. A similar procedure was used to open up the contact pads. Then, the gMμEs were formed by gold electroplating. The photoresist layer was stripped off and a layer of silicon nitride (150 Å)/ silicon oxide (3,000 Å) was deposited by chemical vapor deposition. A third layer of photoresist was then photolithgraphically patterned, followed by wet silicon nitride and silicon oxide etching to selectively remove the silicon nitride and silicone oxide from the contact pads and the mushroom caps. The wafers were then diced and glued to 60 pad printed circuit boards to which a glass ring with a diameter of 20 mm was attached to create a cell culture chamber. Packaged gMμE-MEAs were re-used 3–5 times.

### Skeletal Muscle cultures

Rat skeletal myotubes were cultured following the protocol of Zahavi *et al*.^20^ with minor modifications. Briefly, the hind limb gastrocnemius muscles of newborn rats were incubated in 3 mg/ml collagenase I (C0130, Sigma-Aldrich) in DMEM, 2.5% penicillin-streptomycin-nystatin (PSN) for 1.5 h at 37 °C. The myofibers were then triturated and incubated for 3 days in matrigel coated plates (FAL356234,BD Biosciences) in Bioamf-2 (01-194-1A, Biological Industries) and 1% PSN. To enrich the myoblast population, cells attached to the substrate were trypsinized and replated in plastic dishes for 1 h at 37 °C. Cells that did not adhere to the plastic substrate were transferred to a matrigel-coated dish with bioamf-2 medium. This replating procedure was repeated every day for two to three consecutive days, while keeping the culture at less than 70% confluence. Following the second or third replating cycle the cells were seeded on gold mushroom-shaped matrices or gMμE-MEA functionalized by polydopamin^20^,^70^ and matrigel. The cultures were then maintained at 37 °C and 5% CO2. Half of the culture medium was replaced once every 3 days over a two week period. Electrophysiological data were recorded from the myotubes from day 3 onwards after the last cycle of myoblast enrichment. All procedures were approved by the Committee for Animal Experimentation at the Institute of Life Sciences of the Hebrew University of Jerusalem. All procedures (methods) were carried out in accordance with the approved guidelines.

### Transmission Electron Microscopy

For TEM analysis, skeletal muscle cells cultured on gMμP matrices were fixed, dehydrated and embedded in Agar 100 within the culturing dish as previously described by our laboratory. Briefly, the cells were fixed by 3% glutaraldehyde in a 0.1 M cacodylate buffer (pH = 7.4) for 1 h, at room temperature. The cells were then washed in a 0.1 M cold cacodylate buffer (Agar Scientific, Stansted, UK). Post fixation was done with 1% osmium tetroxide (Next Chimica, Centurion, South Africa) and 0.6% K_3_Fe(CN)_6_ for 1 h. The cells were then washed in a 0.1 M cold cacodylate buffer (pH = 7.4) (Agar Scientific, Stansted, UK). Dehydration was carried out through a series of increasing concentrations of ethanol solutions, and finally the cells were embedded in Agar 100 (Agar Scientific). Then the glass and Ti layer were etched using 39% hydrofluoric acid for approx. 0.5 h. The Au layer was etched by a diluted Au etcher (I_2_/KI/H_2_O), leaving the gold mushroom structures intact. Thereafter, the agar block, including the cells, was re-embedded in Agar 100 in a flat mold. This doubly embedded preparation was then thin-sectioned (70 nm), stained by uranyl acetate and lead citrate, analyzed in TEM Tecnai 12 microscope at 100 kV.

### Electrophysiology

The MEAs used in this study were composed of 8 × 8 gMμEs with a mushroom cap diameter of 2–2.5 μm and a pitch of 100 μm. Altogether the array covered a recording surface of 0.9 × 0.9 mm. Recordings were made using by 60 gMμEs with frequency limits of 1–10000 Hz and a gain of 110–1100. The electrophysiological data shown are unprocessed recordings. Typically, the background noise level of the system was ~20 μV. In all experiments 1–10 ms, 1–10 mV voltage calibration square pulses were applied to the bathing solution by a reference Ag/AgCl electrode using an isolated pulse generator. Conventional intracellular recordings and stimulations of the cultured myotubes were used. The microelectrodes were pulled from 1.5/1.02 mm borosilicate glass tubes with filaments and filled with 2 M KCl. Electrode resistance ranged between 4 and 10 MΩ.

### Modeling

The simulations of a single or electrically coupled myotube were conducted using Tanner EDA V15.0 that relies on the SPICE environment. Calculations and graph presentations used MATLAB (2014A). The simulations were conducted in the following manner.

The passive membrane properties of a single myotube was simulated by an analog electrical circuit composed of a resistor (R) and capacitor (C) in parallel (R = 100 MΩ and C = 100pF) (Fig. 8ai). Two coupled myotubes were simulated by two analog circuits R_1_C_1_ and R_2_ C_2_ (R_1_ = R_2_ and C_1_ = C_2_) connected by a coupling resistor R_c_ = 250 MΩ (Fig. 8bi). The spread of action potentials along two electrically coupled cells and the contribution of a neighboring cell to the electrical load on a firing cell were studied in the following way. First we fed a current pulse with a myotube-spike-waveform shape into an analog circuit depicting a single myotube (Fig. 8ai). The injected current intensity was adjusted to generate an 80 mV “action potential” across the RC circuit depicting the plasma membrane of a single myotube.

The spread of an action potential generated in cell 1 to cell 2 was monitored across the RC circuit of cell 2 when cell 1 and 2 were coupled by the coupling resistor R_c_. To measure the impact of the load of cell 2 on the amplitude of the action potential generated by cell 1 (in cell 1), we measured the “action potential” amplitude in cell 1 using the current intensity that was established to generate an 80 mV action potential in the isolated cell.

To simulate the effects of simultaneous or near simultaneous firing of “action potentials” on the spikes’ amplitudes, action potentials were generated (by current injection) in the two cells at different time intervals.

### Coupling of myotubes to a gMμE

To assess the expected coupling coefficient between cultured muscle fibers and a gMμE we simulated the electrical coupling between coupled myotubes and a gMμE (Fig. 8d).

For this simulation the cleft formed between the plasma membrane and a gMμE (Fig. 8d) was represented by a resistor (Rs), taking into account the medium’s specific resistance, electrical dynamics, the geometric properties of the membrane and the gMμE^20^,^43^. The gMμE was represented by a constant phase element (CPE) and a resistor in parallel (R_EP_). For the simulations the mushroom shaped protruding structure was constructed of a hemi-ellipsoid shaped cap with a height of 0.5 μm, a diameter of 2 μm and 1 μm-high cylindrical stalks with a diameter of 1 μm. The total surface area of the electrode was calculated to be 9.8 μm^2^, but we used 10 μm^2^ for computational simplicity. The detailed calculations of the gMμE surface area, seal resistance and the membrane and gMμE properties are given in^20^,^43^.

#### Seal resistance (*R*_*s*_)

For the sake of simplicity, the simulations conducted in the present study used a single seal resistance value of 50 MΩ. This value was estimated in an earlier study by Fendyur *et al*.^20^,^48^. It should be noted that experimentally, seal resistance values can vary substantially in the range of 10–100 MΩ (for more details see refs 20,35 and 43).

#### Junctional membrane resistance (*R*_*jm*_)

Using the calculated surface area of the gMμE described above, the corresponding junctional membrane surface area and the junctional membrane resistance and capacitance were estimated. A non-junctional membrane resistance of 100–250 MΩ (*R*_*njm*_) was applied. Naïve calculation of the resistance that should be generated within a junctional membrane area suggested that R_jm_ is >100 GΩ. Earlier studies suggested that the actual resistance of the junctional membrane was significantly smaller. Because of the small surface area of the junctional membrane, *R*_*jm*_ can vary substantially by the recruitment or depletion of single ion channels or by the formation of nanopores due to electroporation or mechanical tension generated at the neuron-electrode interface. In the simulations in the present study we used an *R*_*jm*_ of 600 MΩ (this value was selected to generate a coupling coefficient in the simulation similar to that obtained by the experiments).

The junctional membrane capacitance (*C*_*jm*_) was calculated for a given contact surface area (between the simulated cells and the simulated gMμE) by multiplying the universal value of the specific membrane capacitance (1 μF/cm^2^) and the surface area.

#### gMμE resistance and capacitance

For the simulations we used gMμE depicted by two elements, a constant phase element (*CPE*) and a parallel resistor (*R*_*EP*_) (Fig. 8). The value of the *CPE* impedance was 25 MΩ at 1 KHz, and that of the *R*_*ep*_ was 10 MΩ (for details see refs 20 and 35). An amplifier input capacitance of 8pF and a parallel resistance of 100 GΩ were used in all simulations.

## Additional Information

**How to cite this article**: Rabieh, N. *et al*. On-chip, multisite extracellular and intracellular recordings from primary cultured skeletal myotubes. *Sci. Rep*. **6**, 36498; doi: 10.1038/srep36498 (2016).

**Publisher’s note:** Springer Nature remains neutral with regard to jurisdictional claims in published maps and institutional affiliations.

## Supplementary Material

Supplementary Information
