# Supplementary material for: On-chip, multisite extracellular and intracellular recordings from primary cultured skeletal myotubes
Source: Sci Rep. 2016 Nov 4;6:36498. doi: 10.1038/srep36498 (PMC5095645; doi:10.1038/srep36498)
Supplement: Supplementary Information [file srep36498-s1.pdf]

Supplemental Material

On-chip, multisite extracellular and intracellular recordings from primary cultured skeletal myotubes

Noha Rabieh[1#], Silviya M. Ojovan[1#], Nava Shmoel[1#], Hadas Erez[1], Eilon Maydan[1], and Micha E. Spira[1*]

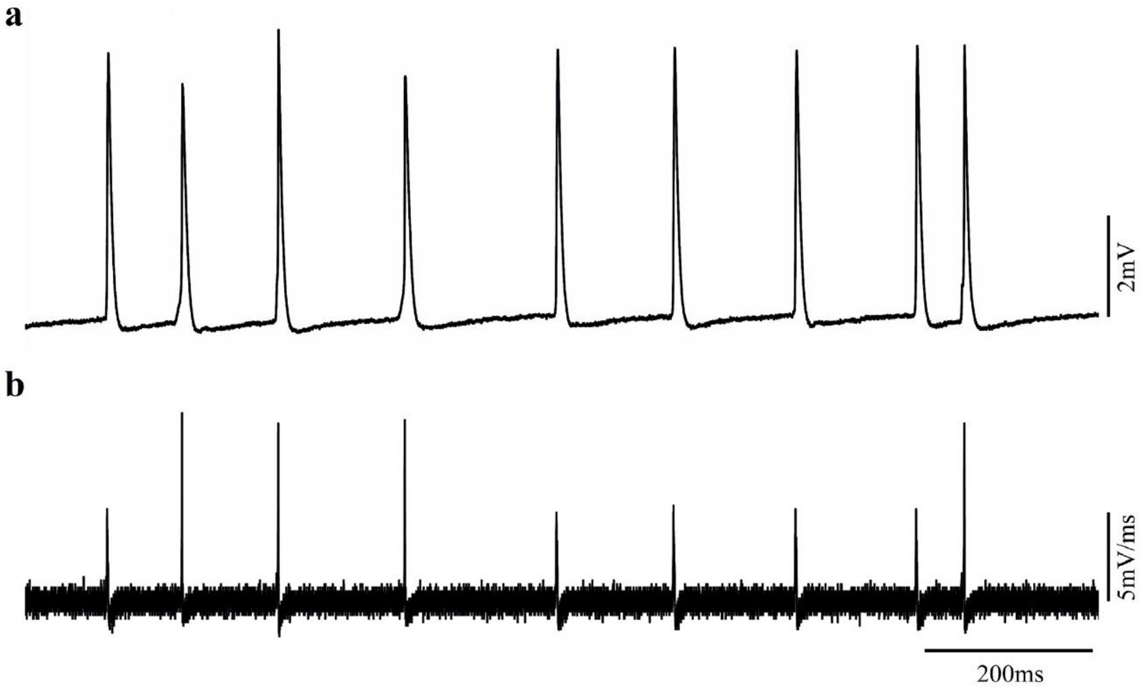

Supplementary Figure 1. Fluctuation in the amplitudes of action potentials as revealed by IN-CELL recordings and their calculated time derivative.
(a) a train of IN-CELL recorded action potentials from a myotube. Note a 10%-20% fluctuations in the APs amplitudes. (b) The amplitude fluctuations of the calculated time derivative are much larger (>50%) than in (a).
